# Cryptic diversity in the Japanese mantis shrimp *Oratosquilla oratoria* (Crustacea: Squillidae): Allopatric diversification, secondary contact and hybridization

**DOI:** 10.1038/s41598-017-02059-7

**Published:** 2017-05-16

**Authors:** Jiao Cheng, Zhong-li Sha

**Affiliations:** Institute of Oceanology, Chinese Academy of Sciences, Qingdao, 266071 China

## Abstract

Mounting evidence of cryptic species in the marine realm emphasizes the necessity to thoroughly revise our current perceptions of marine biodiversity and species distributions. Here, we used mitochondrial cytochrome oxidase subunit I (mtDNA COI) and nuclear ribosomal internal transcribed spacer (nrDNA ITS) to investigate cryptic diversity and potential hybridization in the Japanese mantis shrimp *Oratosquilla oratoria* in the Northwestern (NW) Pacific. Both mitochondrial and nuclear gene genealogies revealed two cryptic species in this morphotaxon, which was further confirmed by extensive population-level analyses. One cryptic species is restricted to cold waters with a distribution range corresponding to temperate affinities, while the other dwelled warm waters influenced by the Kuroshio Current. Their divergence was postulated to be attributable to the vicariant event which resulted from the isolation of the Sea of Japan during the middle Pliocene (*c*. 3.85 Mya, 95% HPD 2.23–6.07 Mya). Allopatric speciation was maintained by limited genetic exchange due to their habitat preferences. Furthermore, the observation of recombinant nrDNA ITS sequence and intra-individual ITS polymorphism suggested recent hybridization event of the two cryptic species occurred in sympatric areas. Our study also illustrated that the Changjiang River outflow might act as an oceanic barrier to gene flow and promoted allopatric diversification in *O. oratoria* species complex.

## Introduction

Marine biodiversity may have always been underestimated due to the occurrence of morphologically indistinguishable cryptic or sibling species^1^. Species has been known to evolve different reproductive and physiological traits without being reflected in the external morphology, adding an extra layer of complexity to the discovery of hidden diversity^2^. The application of molecular methods to species delimitation has uncovered an overwhelming amount of unrecognized cryptic diversity in marine organisms, e.g. copepod^3, 4^, barnacles^5, 6^, bivalves^7, 8^ and fishes^9, 10^. These patterns challenged the paradigm that an apparent homogeneity of the marine environments and pelagic larval stages in marine species limit their diversification^11^. Much progress has been achieved to resolve marine biogeographic patterns and generalize factors involved in shaping marine biodiversity^12, 13^. A growing body of studies has emphasized the importance of climate shifts, or associated sea-level changes caused by glacial cycles, interacted with species-specific ecological traits and life histories as drivers of speciation and diversification in the sea^9, 14–16^.

The Northwestern (NW) Pacific is one of the world’s largest subduction zones, and its shoreline and sea-basin configuration varied extensively and temporarily^17, 18^. Sea levels declined for 120–140 m during the glacial maximum, leading to the complete closure of the Sea of Japan, the semi-closure of the South China Sea and the partial or full exposure of the East China Sea and Yellow Sea^18, 19^. Meanwhile, land bridges were formed between the continent and Taiwan and between the Korean Peninsula and the main Japanese Islands, which would collectively isolate the three marginal seas^18, 20, 21^. During interglacial periods, marine transgression associated with rising sea level caused the coastline of East China Sea-Yellow Sea to move inland, resulting in the flooding of the East China Sea Shelf. The marginal seas reconnected and the islands were isolated again from the continent. These paleogeographical dynamics in the NW Pacific should have enhanced vicariance through glacial geographic isolations and range extension via postglacial dispersal in the marine organisms^9, 22^. This hypothesis has been confirmed by recent phylogeographic investigations in the NW Pacific^16, 23–25^. However, the effects of these evolutionary events in shaping speciation and diversification of marine organisms in this region have not been explored extensively.

**Figure 1:**
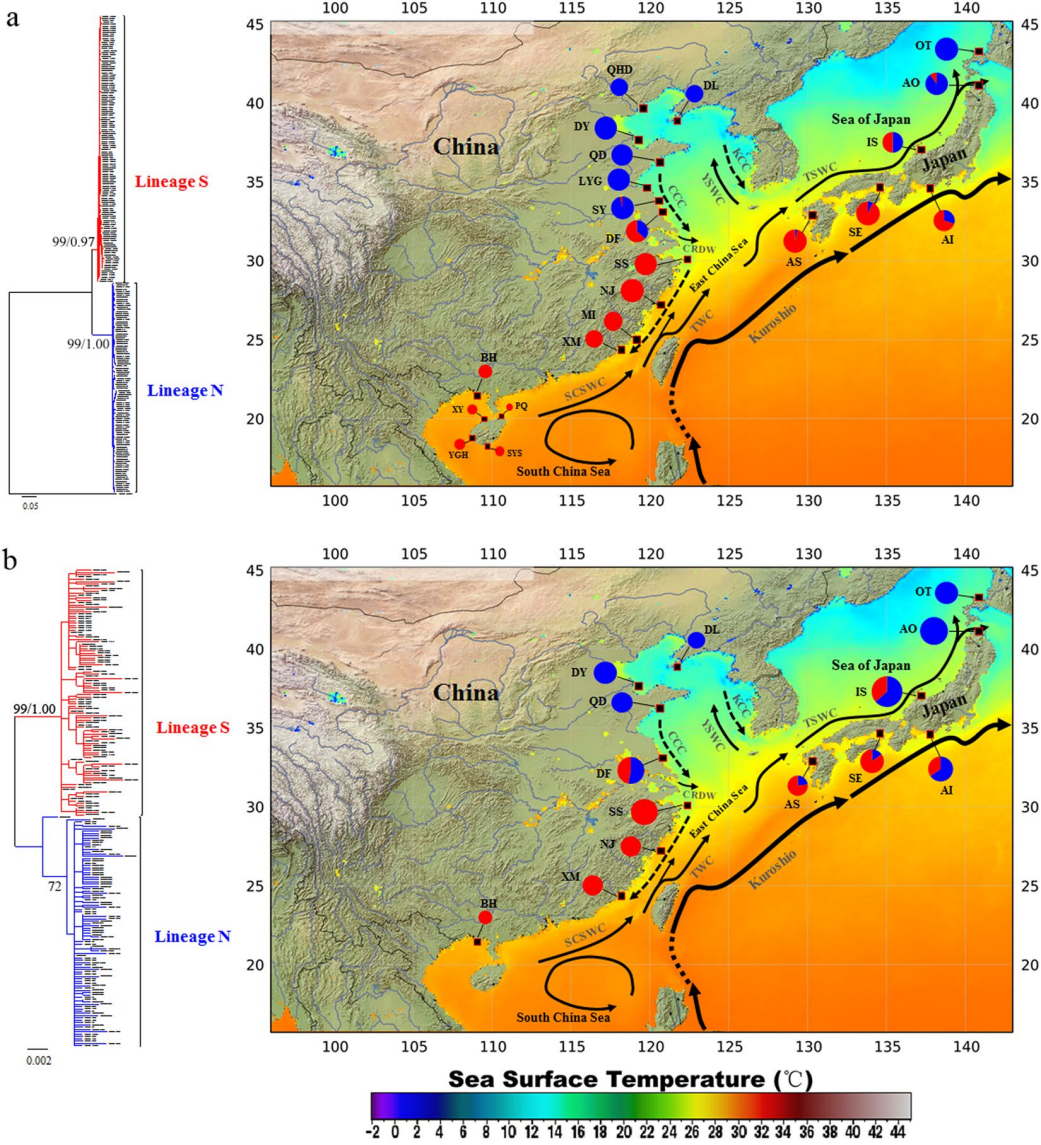
Sample locations and relative frequency of two genetic lineages inferred from mtDNA COI (**a**) and nrDNA ITS (**b**). For each dataset, the corresponding maximum-likelihood (ML) and Bayesian inference (BI) trees are also presented. Numbers above branches represent maximum likelihood bootstrap (left) and Bayesian posterior probabilities (right) for the two major lineages. The map also presented Sea Surface Temperature (SST) in the NW Pacific in winter (2002–2011), which was modified from ref. 78. SCSWC, South China Sea Warm Current; TWC, Taiwan Warm Current; CCC, China Coastal Current; CRDW, Changjiang River Diluted Water; YSWC, Yellow Sea Warm Current; TSWC, Tsushima Warm Current.

Additionally, intricate hydrological systems represented by oceanic currents and freshwater outflow in the NW Pacific may have influenced current population genetic patterns and biogeographic histories of marine species^16, 26–30^. For instance, the Kuroshio Current transports warm and high salinity water northeastward from the Lozon Straits, passes both the east and west coasts of Taiwan and reaches the steep east-west continental shelf break of the East China Sea. It then splits into two branches, the major one flowing northeast towards the southern coast of Kyushu, and the minor one (the Tsushima Warm Current, TSWC) flowing northward into the Sea of Japan^31, 32^. The cold and low-saline China Coastal Current (CCC) flows southwards along the China coast from the Bohai Sea to the Taiwan Strait^33^. The Korean Coastal Current (KCC) connects the East China Sea and the Sea of Japan (Fig. 1). These currents can not only affect dispersal of marine larvae and ultimately population genetic connectivity, but also generate a heterogeneous landscape boundary for marine organisms. Furthermore, the Changjiang River outflow, also known as Changjiang Diluted Water (CDW), has a significant effect on the salinity, nutrient concentrations and planktonic community of the East China Sea^34, 35^. It has been proposed as a physical barrier for genetic arrangements of coastal species inhabiting the region (e.g. *Trachypenaeus curvirostris* ^36^). Thus, the NW Pacific appears to offer an ideal model to disentangle the interaction of contemporary and historical factors driving differentiation and speciation of marine species. One species or a group of closely related species inhabiting marginal seas in the NW Pacific can serve as a model to test these hypotheses.

The Japanese mantis shrimp *Oratosquilla oratoria* (De Haan, 1844) is distributed from Peter the Great Bay, Russia through Japan, Korea and China coastal waters^37, 38^ and exhibits habitat preferences from the shore down to coral reefs and level substrates^39^. Its distribution range spans a wide geographical area throughout tropical, subtropical and temperate coastal areas and covers contrasting environmental features, providing an ideal system to investigate local adaptation and adaptive radiation. *O. oratoria* is subjected to intense and unregulated inshore fisheries in the NW Pacific because of its abundance and wide consumer acceptance, especially in Asian countries^40, 41^. Excessive exploitation has led to severe damage of the natural resources of *O. oratoria* ^42^. Thus, an applicable strategy for conservation and management of this over-exploited species is urgent, and the precise knowledge about its taxonomy and population structure is the prerequisite. Previous studies have revealed a contrasting divergence pattern of *O. oratoria* in China Sea. Zhang *et al*.^43, 44^ found a significant north-south population structure contributed by long-term isolation of the Taiwan Strait during glacial periods. Conversely, Du *et al*.^45^ detected a genetic break between the East China Sea and Yellow Sea which is in accordance with the Changjiang River discharge based on mitochondrial evidence. Nevertheless, these afore-mentioned studies were constrained by small sampling sizes, large geographical gaps in experimental design and inadequate molecular data, which become an impediment to precisely evaluate population genetic structure and cryptic speciation of *O. oratoria*.

Given all uncertainties mentioned above, this study aimed at elucidating a detailed genetic pattern in *O. oratoria* with extensive sampling and revealing the existence of cryptic species. If a species complex did exist, we further attempted to (i) address the driving forces of cryptic speciation; (ii) investigate whether hybridization occurred among cryptic species; (iii) determine the factors influencing current genetic architecture and biogeographic distribution of each cryptic species. To this end, we sequenced mitochondrial cytochrome oxidase subunit I (mtDNA COI) and nuclear ribosomal internal transcribed spacer (nrDNA ITS) sequences from among 22 *O. oratoria* populations across its distribution range. These results can enhance our understanding of how paleoclimate changes and environmental heterogeneity contributed to speciation and diversification of coastal species in the NW Pacific.

## Results

### Phylogeographical structure

#### mtDNA COI

Aligned 658 bp of mtDNA COI gene comprised 144 polymorphic sites which defined 236 haplotypes in 498 specimens. A total of 142 transitions and 16 transversions were scored and no indels were found. All mtDNA COI haplotypes were deposited in GenBank under Accession nos. KY197015–KY197250.

**Figure 2:**
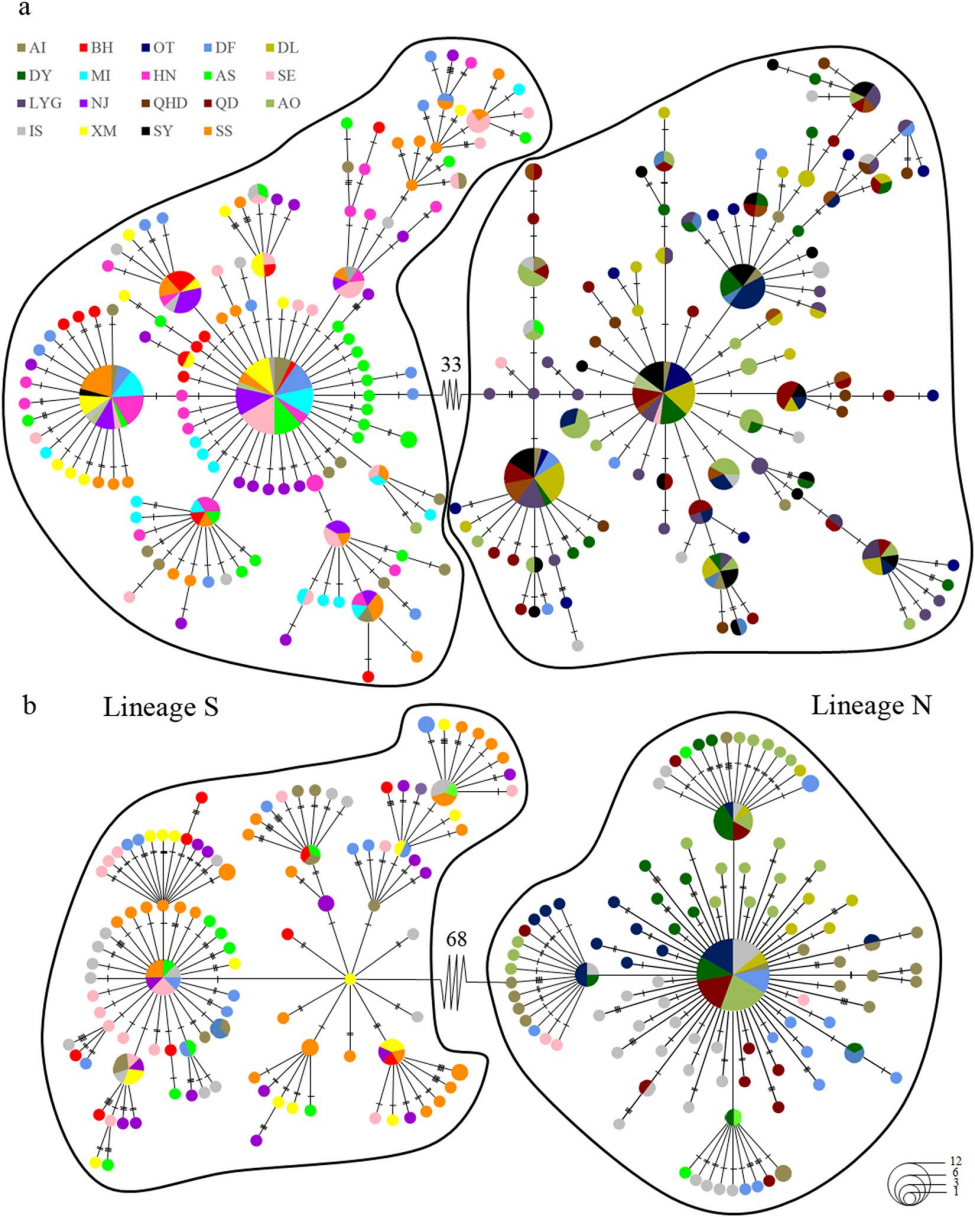
Minimum spanning trees constructed from mtDNA COI (**a**) and nrDNA ITS (**b**) with the sizes of circles proportionally to haplotype/ribotype frequency. The population origins of haplotypes/ribotypes are indicated by colours. Perpendicular tick marks on the lines joining haplotypes/ribotypes represent the number of nucleotide substitutions.

MtDNA COI-based ML and Bayesian trees yielded identical topology and supported two highly-diverged monophyletic lineages in *O. oratoria* (hereafter referred as northern and southern lineages, labelled N and S, Fig. 1a), suggesting two candidate cryptic species. The minimum spanning tree (MST) constructed from mtDNA COI also retrieved two distinct haplogroups. A total of 33 bp of substitutions were required to connect them. For each lineage, common haplotypes were found in the center that produced multiple star-like polytomies with relatively shallow genetic divergences (Fig. 2a). The haplotype network of each lineage indicated no clustering of haplotypes corresponding to localities. The pairwise uncorrected *p*-distances between the two lineages was 6%, whereas the mean genetic distance between haplotypes within each lineage was 0.7% and 0.8%, respectively. Apparently, this satisfied the “4 × rule”^46^ with inter-lineage distances exceeding 4 × intra-lineage distances.

The two divergent mtDNA COI lineages were also geographically structured (Fig. 1a). Overall, lineage N (104 haplotypes, 249 individuals) was dominant in the temperate zone of the NW Pacific, including the northern and central parts of the China coast (the Bohai and Yellow Seas) as well as the northern Japan Sea. By contrast, lineage S (132 haplotypes, 249 individuals) was mainly distributed in subtropical and tropical waters, including the East and South China Seas, and decreased dramatically in frequency along the southern Pacific coast of Japan. These two lineages were sympatric in their range with the southern Yellow Sea and south coast of Japan as overlapped zones (DF, IS and AI).

#### nrDNA ITS

A subset of 147 individuals from 14 populations were genotyped for nrDNA ITS to check the deep phylogenetic break observed by mtDNA COI. After exclusion of regions with mononucleotide and microsatellite repeats, the aligned nrDNA ITS ranged from 1171 bp to 1220 bp and had 307 polymorphic sites which defined 207 ribotypes in 295 clones. Three indels with sizes varying from 4 bp to 48 bp were inferred. According to the simple insertion/deletion coding method^47^, three binary coded gap characters were appended at the end of the combined matrix for phylogenetic analyses. Using the seven algorithms in RDP4, we detected at least one recombination event in nrDNA ITS. Ten of the involved eleven recombinant sequences resembled the ITS1 version of lineage S while ITS2 was concordant to a typical ITS2 version of lineage N, which might be attributed to hybridization succeeded by intralocus recombination in ITS. Recombinant sequences were excluded from subsequent analysis because recombination may produce sequence regions with different evolutionary histories, ultimately impact phylogenies reconstruction and estimations of population genetic parameters^48^. All nrDNA ITS ribotypes were deposited in GenBank under Accession nos. KY197251–KY197457.

NrDNA ITS phylogenies produced concordant lineage structure with mtDNA COI (Fig. 1b), suggestive of two distinctive evolutionary lineages. These two lineages were further supported by the MST (Fig. 2b) which was separated by 68 mutational steps. The MST topology of lineage N was characterized by a star-like polytomy with a dominant haplotype shared by all populations from Japan and northern of China, whereas two star-like polytomies were presented in lineage S. No distinct phylogeographic apportioning of localities was observed in each lineage. For nrDNA ITS, sequence divergence between lineages was 1.6%, and the intra-lineage level of divergence averaged 0.5% and 0.4%, respectively. Surprisingly, intra-individual ITS polymorphism was observed in 17 individuals (Supplementary Table S1), indicating possible past hybridization and introgression between these two evolutionary lineages. Intra-individual sequence divergence of nrDNA ITS was up to 1.8% (within sample AI20, IS11 and IS19), while the maximum intra-individual genetic distance between clones nested in the same lineage was 0.8% (within CS4). A similar pattern of spatial partitioning of ribotypes was observed in nrDNA ITS dataset, with exception that all ribotypes of population AO belonged to lineage N (Fig. 1b).

### Genetic diversity and differentiation of *O. oratoria*

*O. oratoria* populations exhibited high haplotype diversity with overall value of 0.981for mtDNA COI and 0.982 for nrDNA ITS (Table 1). The hybrid populations where two lineages coexisted had higher nucleotide diversity (mtCOI: 0.032–0.037; nrITS: 0.009–0.01) than other populations (mtCOI: 0.003–0.016; nrITS: 0.002–0.008).

**Table 1:** Sample information and molecular diversity indices for *O. oratoria*.

Sample site	Abb	Date of collection	mtCOI					nrITS				
			*N*_*c*_	*n*	*h*	*л*	*L*	*N*_*i*_	*n*	*h*	*л*	*L*
[1] Otaru	OT	2015.06	30	20	0.947 ± 0.027	0.005 ± 0.003	N	18	12	0.895 ± 0.065	0.002 ± 0.001	N
[2] Aomori	AO	2015.10	30	18	0.959 ± 0.018	0.016 ± 0.008	N, S	27	19	0.917 ± 0.047	0.002 ± 0.001	N
[3] Qinhuangdao	QHD	2014.05	20	18	0.984 ± 0.024	0.006 ± 0.004	N					
[4] Dalian	DL	2014.05	30	17	0.933 ± 0.028	0.005 ± 0.003	N	8	7	0.964 ± 0.077	0.002 ± 0.001	N
[5] Dongying	DY	2014.11	23	17	0.961 ± 0.027	0.005 ± 0.003	N	19	12	0.906 ± 0.050	0.002 ± 0.001	N
[6] Qingdao	QD	2014.05	30	23	0.977 ± 0.015	0.005 ± 0.003	N	16	10	0.867 ± 0.079	0.002 ± 0.001	N
[7] Lianyungang	LYG	2015.09	30	23	0.977 ± 0.016	0.005 ± 0.003	N					
[8] Sheyang	SY	2015.05	30	20	0.961 ± 0.020	0.009 ± 0.005	N, S					
[9] Dafeng	DF	2015.05	30	24	0.972 ± 0.021	0.035 ± 0.018	N, S	32	25	0.980 ± 0.015	0.010 ± 0.005	N, S
[10] Ishikawa	IS	2013.10	20	18	0.990 ± 0.019	0.037 ± 0.019	N, S	44	39	0.988 ± 0.010	0.009 ± 0.005	N, S
[11] Aichi	AI	2015.10	20	18	0.984 ± 0.024	0.032 ± 0.016	N, S	26	24	0.994 ± 0.013	0.010 ± 0.005	N, S
[12] Setonaikai	SE	2015.10	30	19	0.936 ± 0.032	0.013 ± 0.007	N, S	20	19	0.995 ± 0.018	0.007 ± 0.004	N, S
[13] Ariake Sea	AS	2015.02	30	25	0.975 ± 0.021	0.010 ± 0.005	N, S	13	13	1.000 ± 0.030	0.008 ± 0.005	N, S
[14] Shengshan	SS	2015.07	30	23	0.961 ± 0.027	0.007 ± 0.004	S	31	26	0.989 ± 0.011	0.005 ± 0.003	S
[15] Nanji	NJ	2014.06	30	20	0.954 ± 0.023	0.006 ± 0.003	S	16	15	0.992 ± 0.025	0.004 ± 0.003	S
[16] Meizhou Island	MI	2014.04	22	15	0.931 ± 0.040	0.005 ± 0.003	S					
[17] Xiamen	XM	2014.04	20	13	0.926 ± 0.043	0.005 ± 0.003	S	16	14	0.983 ± 0.028	0.004 ± 0.002	S
[18] Beihai	BH	2015.07	16	14	0.975 ± 0.035	0.006 ± 0.003	S	9	9	1.000 ± 0.052	0.006 ± 0.003	S
[19] Xinying	XY	2007.11	10	9	0.978 ± 0.054	0.006 ± 0.004	S					
[20] Puqian	PQ	2007.12	3	2	0.677 ± 0.314	0.003 ± 0.003	S					
[21] Yinggehai	YGH	2007.12	8	8	1.000 ± 0.063	0.004 ± 0.003	S					
[22] Sanya	SYS	2008.03	6	5	0.933 ± 0.122	0.006 ± 0.004	S					
Total			498	236	0.981 ± 0.002	0.032 ± 0.016		295	207	0.982 ± 0.005	0.032 ± 0.015	

Abb., abbreviation of locality; *N*_*c*_, number of sequenced individuals; *n*, number of haplotypes; *N*_*i*_, number of clones; *h*, haplotype diversity; *л*, nucleotide diversity; *L*, lineage found in each locality.

Based on the distribution of haplotypes, we defined two distribution regions for the sampled *O. oratoria* populations: (T) temperate region (including the Bohai and Yellow Seas as well as the northern Japan Sea; site1–10), and (S) subtropical and tropical region (including the East and South China Seas as well as the southern Pacific coast of Japan; site 11–22). Pairwise *F*_ST_ values between populations of different regions were high (0.749–0.928 for mtDNA COI; 0.745–0.801 for nrDNA ITS) and statistically significant after Bonferroni corrections for both markers (Table 2). Moderate and significant *F*_ST_ values (0.127–0.653 for mtDNA COI; 0.094–0.39 for nrDNA ITS) were also observed for pairwise comparisons between hybrid populations (site 9–11) and other populations. In addition, most of the pairwise *F*_ST_ values between populations within each region were low and statistically non-significant. Further analysis by hierarchical AMOVA indicated that for mtDNA COI 72.32% of genetic variation was partitioned by regions, while for nrDNA ITS 38.72% of genetic variation occurred among regions (Table 3). An AMOVA analysis that excluded the hybrid populations showed more suitable variance partitioning with higher percentage of variance (88.24% for mtDNA COI, 71.28% for nrDNA ITS) among regions (P < 0.001, Table 3). Genetic subdivision was highly significant between regions (*F*_CT_ = 0.882, *P* < 0.001 for mtCOI; *F*_CT_ = 0.713, *P* < 0.001 for nrITS), indicating a high level of geographical population structure.

**Table 2:** Pairwise *F*_ST_ comparisons between populations inferred from mtDNA COI (below diagonal) and nrDNA ITS (above diagonal).

	[1]	[2]	[3]	[4]	[5]	[6]	[7]	[8]	[9]	[10]	[11]	[12]	[13]	[14]	[15]	[16]	[17]	[18]
[1] OT	—	0.017		0.041	0.047^*^	0.017			0.254 ^**^	0.159 ^**^	0.172 ^*^	**0.543** ^**^	**0.549** ^**^	**0.745** ^**^	**0.792** ^**^		**0.801** ^**^	**0.792** ^**^
[2] AO	0.041	—		−0.016	−0.004	−0.010			0.286 ^**^	0.185 ^**^	0.210 ^**^	**0.572** ^**^	**0.577** ^**^	**0.755** ^**^	**0.791** ^**^		**0.799** ^**^	**0.792** ^**^
[3] QHD	−0.002	0.029	—															
[4] DL	0.019	0.036	0.004	—	−0.020	−0.028			0.214 ^*^	0.133 ^*^	0.144 ^*^	**0.499** ^**^	**0.475** ^**^	**0.721** ^**^	**0.760** ^**^		**0.772** ^**^	**0.743** ^**^
[5] DY	−0.012	0.033	−0.001	0.009	—	−0.002			0.264 ^**^	0.171 ^*^	0.192 ^**^	**0.546** ^**^	**0.542** ^**^	**0.741** ^**^	**0.778** ^**^		**0.788** ^**^	**0.774** ^**^
[6] QD	0.025^*^	0.036	−0.006	−0.009	0.02	—			0.247 ^**^	0.156 ^*^	0.175 ^**^	**0.536** ^**^	**0.531** ^**^	**0.738** ^**^	**0.779** ^**^		**0.789** ^**^	**0.775** ^**^
[7] LYG	0.017	0.033	−0.001	0.004	0.008	0.002	—											
[8] SY	0.001	−0.001	−0.004	−0.004	−0.013	−0.004	−0.01	—										
[9] DF	0.542 ^**^	0.400 ^**^	0.492 ^**^	0.543 ^**^	0.508 ^**^	0.540 ^**^	0.530 ^**^	0.487 ^**^	—	0.000	−0.002	0.094 ^**^	0.053	0.309 ^**^	0.275 ^**^		0.290 ^**^	0.254 ^**^
[10] IS	0.461 ^**^	0.287 ^**^	0.400 ^**^	0.463 ^**^	0.420 ^**^	0.459 ^**^	0.447 ^**^	0.392 ^**^	−0.011	—	−0.008	0.176 ^**^	0.124 ^*^	0.390 ^**^	0.366 ^**^		0.380 ^**^	0.348 ^**^
[11] AI	0.652 ^**^	0.492 ^**^	0.599 ^**^	0.653 ^**^	0.617 ^**^	0.648 ^**^	0.639 ^**^	0.591 ^**^	−0.029	0.020	—	0.156 ^**^	0.109 ^*^	0.379 ^**^	0.348 ^**^		0.364 ^**^	0.320 ^**^
[12] SE	**0.856** ^**^	**0.749** ^**^	**0.835** ^**^	**0.857** ^**^	**0.842** ^**^	**0.852** ^**^	**0.848** ^**^	**0.817** ^**^	0.179 ^*^	0.320 ^**^	0.127 ^**^	—	−0.025	0.101^**^	0.062^*^		0.079^*^	0.045
[13] AS	**0.886** ^**^	**0.783** ^**^	**0.871** ^**^	**0.888** ^**^	**0.877** ^**^	**0.883** ^**^	**0.879** ^**^	**0.849** ^**^	0.222 ^**^	0.371 ^**^	0.170 ^**^	−0.003	—	0.137^**^	0.103^**^		0.123^**^	0.068
[14] SS	**0.913** ^**^	**0.814** ^**^	**0.901** ^**^	**0.914** ^**^	**0.906** ^**^	**0.909** ^**^	**0.906** ^**^	**0.877** ^**^	0.265 ^**^	0.426 ^**^	0.230 ^**^	0.007	0.021^*^	—	0.013		0.016	−0.007
[15] NJ	**0.920** ^**^	**0.822** ^**^	**0.910** ^**^	**0.921** ^**^	**0.914** ^**^	**0.916** ^**^	**0.914** ^**^	**0.885** ^**^	0.274 ^**^	0.435 ^**^	0.237 ^**^	0.019	0.002	0.014	—		−0.028	−0.016
[16] MI	**0.927** ^**^	**0.817** ^**^	**0.917** ^**^	**0.928** ^**^	**0.921** ^**^	**0.922** ^**^	**0.920** ^**^	**0.887** ^**^	0.255 ^*^	0.412 ^**^	0.219 ^**^	0.015	−0.001	−0.009	−0.008	—		
[17] XM	**0.924** ^**^	**0.810** ^**^	**0.913** ^**^	**0.926** ^**^	**0.918** ^**^	**0.920** ^**^	**0.917** ^**^	**0.883** ^**^	0.244 ^**^	0.394 ^**^	0.212 ^**^	0.036	0.012	0.024	0.011	0.008	—	−0.007
[18] BH	**0.923** ^**^	**0.800** ^**^	**0.910** ^**^	**0.925** ^**^	**0.916** ^**^	**0.918** ^**^	**0.915** ^**^	**0.878** ^**^	0.221 ^**^	0.366 ^**^	0.182 ^*^	0.011	−0.012	0.004	−0.021	−0.015	−0.011	—
[19–22] HN	**0.924** ^**^	**0.821** ^**^	**0.914** ^**^	**0.925** ^**^	**0.918** ^**^	**0.919** ^**^	**0.917** ^**^	**0.887** ^**^	0.265 ^**^	0.428 ^**^	0.229 ^**^	0.019	0.009	−0.014	−0.001	−0.018	0.015	−0.015

^*^ *P* < 0.05; ^**^ *P* < 0.001.

**Table 3:** Results of analysis of molecular variance (AMOVA) for different hierarchical analyses of *O. oratoria* populations.

Structure tested	mtDNA COI				nrDNA ITS			
	df	Variance	%total	*F* statistics	df	Variance	%total	*F* statistics
Two gene pools	(Group I: 1–10; II: 11–22)				(Group I: 1–2, 4–6, 9–10; II: 11–15, 17–18)			
Among regions	1	18.35	72.32	*F*_CT_ = 0.723^**^	1	2.79	38.72	*F*_CT_ = 0.387^*^
Among populations with region	16	1.32	7.15	*F*_SC_ = 0.258^**^	12	0.81	11.26	*F*_SC_ = 0.184^*^
Wthin populations	450	3.79	20.53	*F*_ST_ = 0.795^**^	281	3.60	50.02	*F*_ST_ = 0.500^**^
Two gene pools	(Group I: 1–8; II: 12–22)				(Group I: 1–2, 4–6; II: 12–15, 17–18)			
Among regions	1	18.55	88.24	*F*_CT_ = 0.882^**^	1	6.34	71.28	*F*_CT_ = 0.713^**^
Among populations with region	13	0.02	0.12	*F*_SC_ = 0.010^*^	9	0.07	0.83	*F*_SC_ = 0.029^*^
Wthin populations	383	2.45	11.64	*F*_ST_ = 0.884^**^	182	2.48	27.89	*F*_ST_ = 0.721^**^

^*^ *P* < 0.05; ^**^ *P* < 0.001.

### Divergence between lineages

The pattern of reciprocal monophyly, deep phylogenetic divergence between lineages and limited genetic exchange between populations detected in *O. oratoria* indicated the existence of two cryptic species across its distribution. For each location, individuals belonging to the same lineage were grouped together to further explore the genetic structure of the two cryptic species. Population genetic differentiation between lineages was highly significant (*F*_ST_ = 0.916, *P* < 0.001 for mtCOI; *F*_ST_ = 0.722, *P* < 0.001 for nrITS). No genetic heterogeneity was detected among samples assigned to each of these two lineages (Supplementary Table S2). However, the generality of these results may be limited by the relatively low sample sizes of AI-N, SE-N and AS-N when compared with populations with a larger sample size. The calibrated divergence time between the two lineages was 3.85 Million Years ago (Mya) (95% HPD 2.23–6.07 Mya), dating back to the middle Pliocene. The coalescence time was estimated at 0.6 Mya (95% HPD 0.33–0.87 Mya) and 0.62 Mya (95% HPD 0.36–0.92 Mya) for lineage N and lineage S, respectively.

### Historical demography

**Figure 3:**
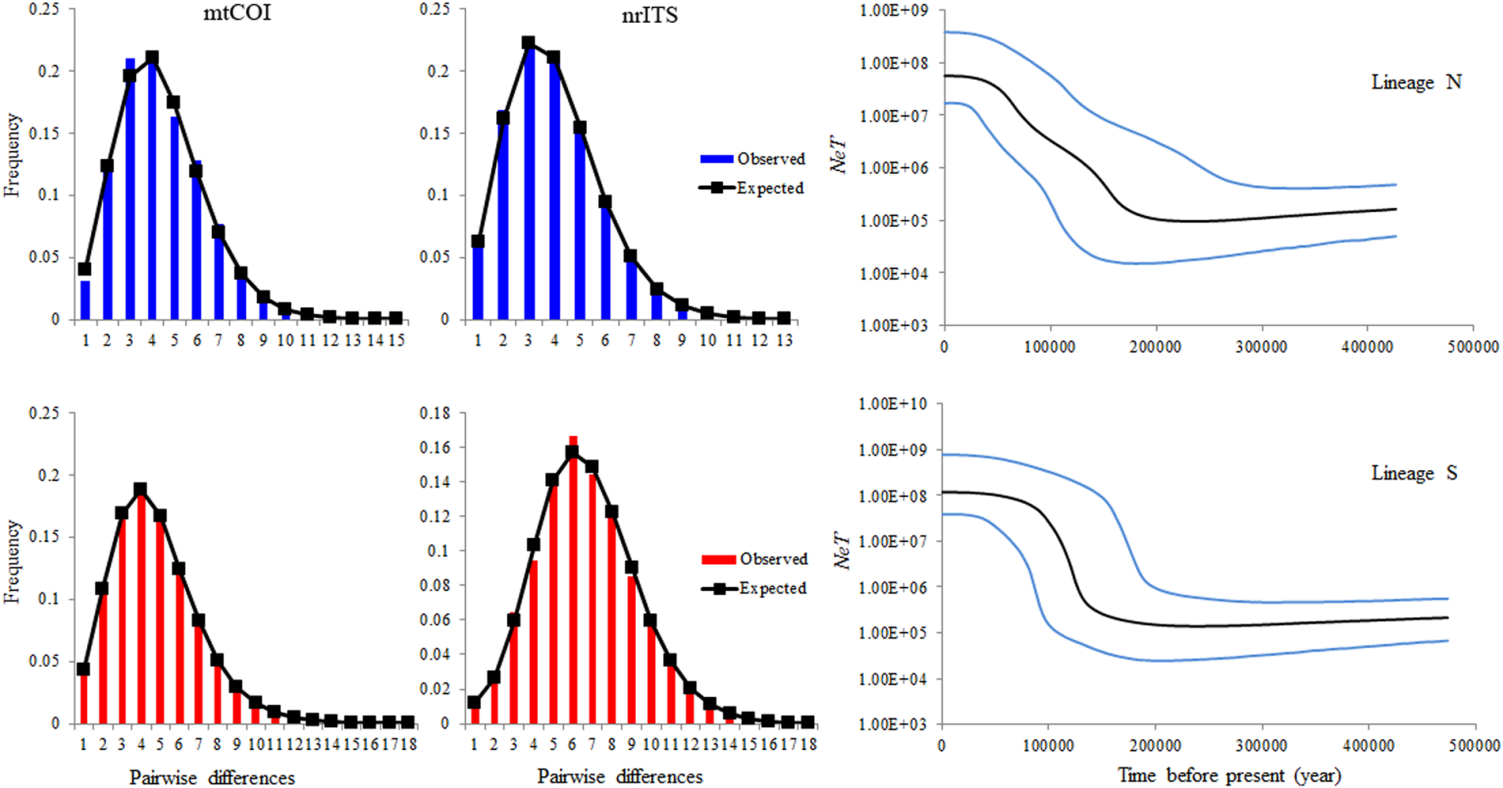
Mismatch distributions and Bayesian skyline plots for each genetic lineage. In mismatch distributions, bars represent the observed frequency of pairwise differences, whereas the solid lines show the expected values under the sudden expansion model. The BSPs show *N*_*e*_ *T* (*N*_*e*_ = effective population size; *T* = generation time) changes through time. Black lines represent median estimates while the blue lines show the 95% highest posterior density (HPD) limits.

Tajima’s *D* and Fu’s *F*_S_ calculated for each lineage were negative and statistically significant (Table 4), indicating recent demographic expansions. The sequential mismatch distributions of the two lineages (Fig. 3) and the permutation tests with the SSD and HRI statistics (Table 4) all suggested a unimodal distribution, providing further evidence of population expansion. BSP analysis indicated that two lineages have experienced exponential demographic increases in the late Pleistocene. Specifically, lineage N experienced expansion at *c*. 200 000 years ago, whereas lineage S was dramatic in its increase in both rate and timing and showed a more recent population expansion at *c*. 180 000 years ago (Fig. 3).

**Table 4:** Summary of molecular diversity and demographic analyses for two lineages of *O. oratoria*.

Groups	*N*	*n*	*h*	*л*	Tajima’s *D*		Fu’s *Fs*		Mismatch distribution			
					*D*	*P*	*Fs*	*P*	*θ*_*0*_	*θ*_*1*_	*P*(SSD)	*P*(HRI)
**mtCOI**												
Lineage N	249	104	0.969 ± 0.005	0.005 ± 0.003	−2.119	0.000	−25.842	0.000	0.689	9999	0.376	0.548
Lineage S	249	132	0.954 ± 0.008	0.006 ± 0.003	−2.345	0.000	−25.654	0.000	1.182	68.672	0.965	0.727
**nrITS**												
Lineage N	156	100	0.941±0.015	0.003 ± 0.001	−2.865	0.000	−26.409	0.000	0.682	271.895	0.910	0.676
Lineage S	139	107	0.992 ± 0.003	0.005 ± 0.002	−2.650	0.000	−25.135	0.000	0.796	121.719	0.782	0.689

Number of individuals (*N*), number of haplotypes or ribotypes (*n*), haplotype diversity (*h*), nucleotide diversity (*л*), Tajima’s *D* and Fu’s *F*_S_ with corresponding P values for each lineage are shown. *θ*_0_ and *θ*_1_ are *θ* parameter before and after expansion; *P* values for the sum of squared deviations (SSD) and raggedness index (HRI) under the hypothesis of sudden expansion of each lineage are also shown.

## Discussion

### Allopatric diversification and biogeographic processes

Two major genealogical lineages and a strong phylogeographical structure were detected in our mtDNA analyses of *O. oratoria*. The two divergent lineages may represent vicariant relicts of an ancestral population which was isolated in two allopatric areas during the middle Pliocene. The geographical distribution of lineage S implies an origin in the South China Sea. Although it is difficult to identify potential origin for lineage N due to the lack of samples from Okinawa, its broad distribution in the Bohai Sea, Yellow Sea and northern Japan Sea, in combination with the dated ages concur in suggesting a possible origin in the Sea of Japan. As a composite marginal sea in the NW pacific, the Sea of Japan is a semi-enclosed sea and it connects with nearby water bodies through narrow straits that are shallow and less than 130 m deep. During the middle Pliocene, the Sea of Japan was strongly influenced by a cold northern surface water until 3.5 Mya when the warm Tsushima Current first reappeared corresponding to the reopening of the Tsushima Strait^49, 50^. Meanwhile, the South China Sea was isolated from the East China Sea by landmasses formed between continental China and Taiwan^51^. This process may have been the powerful vicariant event for allopatric differentiation of the ancestral *O. oratoria* populations. During each glacial episode, ancestors of the lineage N that were trapped in the Sea of Japan probably evolved to adapt in cold water environment. By contrast, relatively high-temperature adaptation may have evolved in ancestors of lineage S. In reality, similar phylogeographic pattern was also observed in several other marine species in this region, such as two varieties of *Sargassum* ^52^, flathead mullet *Mugil cephalus* ^9^ and the bivalve *Cyclina sinensis* ^24^, although the magnitude of genetic subdivision varies among species. Taken together, complex coastal topography and habitat patchiness in the NW Pacific driven by the recurrent drops in sea level over the past few million years probably structured biogeographic shifts of marine organisms at various temporal and spatial scales^53^.

Several of our statistical analyses yielded signatures of major demographic expansions for both lineages in *O. oratoria* in the late Pleistocene (Table 4, Fig. 3). Glacial-interglacial climate fluctuations during the Pleistocene epoch led to changes of sea level and then caused habitat contractions or expansions, which might have reinforced ancient vicariance and postglacial dispersal of *O. oratoria*. The rising sea-level and temperature driven by climate changes during the interglacial period might have brought drastic events of postglacial marine transgression in the Yellow and East China seas due to the shallow continental shelves^18, 54^, generating large amounts of shallow coastal habitat for *O. oratoria*. In this context, demographic expansions and range extension of ancestral *O. oratoria* populations would have accompanied a series of colonizing events in previously unoccupied habitats. Specifically, the Bohai and Yellow Seas were colonized by lineage N from a northern route of dispersal through the Tsushima Strait, whereas the East China Sea and the Pacific coast of Japan were recolonized by lineage S from southern route that followed the Kuroshio Current. Lastly, sympatric distribution of two lineages in the Sea of Japan might ascribe to secondary contact sometime after a geographically northward expansion or range shifts of lineage S that was facilitated by the Tsushima Current. This scenario is also supported by the observation that warm-water planktonic foraminifera and mollusks migrated into the Sea of Japan under the same effects of the Tsushima Current^55^. Although glacial influence to population fluctuation was observed in late Pleistocene, it seems population size of *O. oratoria* was persistent through the Last Glacial Maximum (LGM, 26 000–19 000 years ago). Previous paleoclimate evidence demonstrated that the most substantial global glacial extension occurred in the Marine Isotope Stages 16–18 (MIS 16-MIS 18, 0.6–0.7 Mya)^56, 57^. Since then, environmental changes seem to be moderate in subsequent climate oscillations. Recent analyses of demographic history of marine invertebrate species in the NW Pacific^58, 59^ also converged on similar findings that majority of the species had potential abilities to survive the LGM and regional persistence maybe more prevalent.

### Cryptic species and hybridization in *O. oratoria* complex

Two reciprocally monophyletic, highly supported lineages together with large inter-lineage relative to intra-lineage divergences recovered from mtDNA COI data allowed us to propose the existence of two candidate cryptic species under the “4 × rule” for species detection^46^. Although initially designed for asexual organisms, the “4 × rule” can also be applied to mitochondrial genes in sexual organisms and has become a promising tool for cryptic species delimitation in crustaceans^60, 61^. In this study, the divergence level between *O. oratoria* lineages (6%) is comparable to that calculated between some cryptic species of stomatopods species (*Haptosquilla pulchella*: 2.9–6.6%; *Haptosquilla glyptocercus*: 4.7–11.7%; *Gonodactylellus viridis*: 3.0%–10.6%^62^) or barnacle species (*Chthamalus moro*: 3.9–8.3%^6^). This COI divergence value also exceeded the COI species screening threshold (5% divergence) applied for stomatopods^63^, a value also supported by data from alpheid shrimp in which most sister species showed more than 5% sequence divergence^49^. Nuclear evidence of two monophyletic lineages in combination with a relatively long period of divergence between two *O. oratoria* lineages (since middle Pliocene) concur in suggesting that lineage sorting of ancestral polymorphisms has been completed. Moreover, the clear allopatric distribution of the two lineages also tend to support the presence of cryptic species: lineage N was almost exclusively temperate whereas lineage S was mostly tropical and subtropical and extended into temperate regions of Japan despite being found in sympatry at their geographical boundaries. Lastly, further evidence for species distinctiveness is that the gene flow appeared to be more limited among sympatric individuals belonging to different lineages than between geographically distant individuals sharing the same lineage (Supplementary Table S2). Given the marked genetic isolation and limited gene flow between natural populations of lineage N and S as revealed from evidence of both mitochondrial and nuclear gene, we argue that they could actually constitute different species.

Natural hybridization occurs relatively frequently when previously allopatric populations or species achieve secondary contact^64^. Unless reproductive isolation is complete, any secondary contact would be followed by hybridization^65, 66^. In this study, we identified species-specific nuclear ribotypes as they are highly divergent and exclusively present in allopatric populations of one cryptic species. Presence of such species-specific alleles in an individual genome reflected either a past hybridization or a gene duplication event^67^. For nrDNA ITS sequencing, approximately half samples were sequenced with more than two clones, and most of them were represented by more than two alleles, indicating that the intragenomic variance observed in *O. oratoria* could not be an occasional gene duplication event. Instead, the observation of recombinant nrDNA ITS sequence, intra-individual polymorphism of nrDNA ITS in 17 individuals and hybrids sharing their nrDNA ITS ribotypes with nonhybridization individuals all suggested recent past hybridization has occurred. However, absence of morphological divergence between the two *O. oratoria* cryptic species makes it difficult to decipher the direction and magnitude of introgression. In our study, most putative hybrids have been observed to possess two types of nrDNA ITS ribotypes, implying bidirectional nuclear introgression. The three hybrids in the population AO showed an exceptional trend; all their nrDNA ITS ribotypes (28 clones in total) belonged to lineage N, but their mtDNA COI sequences were all grouped into lineage S (Supplementary Table S1). A reasonable explanation was the mitochondrial introgression from lineages S to N. Further studies are warranted to address the rate and pattern of hybridization, which in return can offer further insights into the origin and driving forces of hybridization between *O. oratoria* species complex.

### Population structure and distribution pattern

Recent studies reported different pattern of genetic differentiation of *O. oratoria* populations in China Sea and identified two genetic groups as a consequence of long-term isolation by the Taiwan Strait^43, 44^. Instead, our study detected a sharp genetic discontinuity among China coastal populations in line with the Changjiang Estuary, a finding generally consistent with the mtDNA analysis by Du *et al*.^45^. In the sea, it is well recognized that the movements of pelagic stages are directly influenced by ocean currents that can lead to dispersal of hundreds of kilometres^68, 69^. As benthic habitat for adults, genetic exchanges among *O. oratoria* populations mainly occur in the planktonic larval stage. Laboratory-reared larvae of *O. oratoria* have a planktonic stage of between 36 and 59 days^70^, indicating high potential for dispersal during its planktonic larval stage. Thus, the lack of genetic structure in each *O. oratoria* cryptic species is not surprising because oceanographic current systems in the NW Pacific are expected to facilitate larvae dispersal over considerable distances, eventually homogenizing widely spaced coastal populations. On the other hand, dynamic oceanographic processes, such as freshwater outflow, local gyres, upwelling systems and fronts, can also profoundly restricted connectivity among populations. Mounting genetic evidence shows that limited connectivity was found in coastal species despite long-lived pelagic larvae, which generally arise from restricted oceanographic exchange among geographically proximate locations^29, 71, 72^. The Changjiang River outflow influences its surrounding hydrological condition and causes a decline in the salinity of the upper layer of the Kuroshio Current^73^, which may have some impacts on gene flow between the Yellow Sea and East China Sea. For *O. oratoria*, limited larval transport influenced by the Changjiang River outflow may act as a mechanism promoting allopatric diversification, and may explain the observed genetic discontinuity along the China Coast. Similar genetic breaks that resulted from the Changjiang River freshwater discharge have been reported in some other marine species, including crustacean *Trachypenaeus curvirostris* ^36^, macroalga *Sargassum hemiphyllum* ^52^ and gastropod *Cellana toreuma* ^74^. However, the barrier effect of the Changjiang River outflow on gene flow of costal species might have weakened as a consequence of global warming^59^. The present-day occurrence of lineage S in the southern of Yellow Sea probably results from its northward expansion as the frontal zone between the Kuroshio Current and the Changjiang River outflow shifts northwards according to the strength of the Kuroshio Current^75, 76^.

A well-characterized natural environmental gradient in the NW Pacific is its steep thermal cline. The distributional ranges of the two *O. oratoria* cryptic species closely parallel the spatial distribution pattern of Sea Surface Temperature (SST) in the NW Pacific governed by the oceanic currents system in this region (Fig. 1). The temperature species inhabits regions influenced by cold China Costal Current and Korean Coastal Current which facilitate cold water flowing southward from the Yellow Sea with average of water temperature from 12 °C in March to 20 °C in September^77^. By contrast, the distribution of the subtropical and tropical species corresponds to the route of the warm Kuroshio Current and its branches. The Kuroshio Current remains warm throughout the year (23–26 °C), and facilitates a great number of warm-water marine species migrate northward from their tropical center and expand their distribution ranges^78^. The strong correlation between species distributions and flows of oceanographic currents associate with their temperatures implies an adaptation of *O. oratoria* species complex to local thermal regimes. This finding is not unusual because recent genetic studies of *M. cephalus* species complex revealed that water temperature is one of the main factors that affect the distribution of three NW Pacific cryptic species^9^. Such close relationships between oceanic currents system and geographical distribution of genetic variation were also observed in the king weakfish, *Macrodon ancylodon*, along Atlantic coastal waters of South America^14^. Further researches on adaptive selection are necessary to better understand the role of temperature gradients in the NW Pacific in generating species boundaries and in shaping the contemporary spatial distribution of *O. oratoria* species complex, and their adaption to local environment.

## Conclusions

The physical and environmental attributes of the Northwestern (NW) Pacific provide an ideal model to explore mechanisms underpinning speciation and evolutionary processes of marine species. Our results revealed cryptic speciation in the Japanese mantis shrimp *O. oratoria* by integrating mitochondrial and nuclear evidence. These two cryptic species likely diverged in allopatry during the middle Pliocene epoch, followed by range extensions and secondary contact that resulted in their present-day distribution. Our analyses also revealed recent hybridization event of the two cryptic species occurred in sympatric areas. The distribution range of the two *O. oratoria* cryptic species exhibits a remarkable latitudinal cline, which might be correlated to distinct temperature preferences of the species. This study highlights the interactive role of paleoclimate changes and environmental heterogeneity in driving genetic diversification of coastal species in the NW Pacific and underscores the importance of phylogeographic data in revealing cryptic marine biodiversity. From a conservation perspective, management efforts will need to maintain the genetic integrity and evolutionary potential of each cryptic species in their native regions.

## Methods

The methods were carried out in accordance with the approved guidelines of the Good Experimental Practices adopted by Institute of Oceanology, Chinese Academy of Sciences. All experimental protocols were conducted under the permits approved by Institute of Oceanology, Chinese Academy of Sciences, China.

### Sampling, sequencing and alignment

A total of 498 *O. oratoria* individuals were collected from 22 localities along the coast of China and Japan (Fig. 1, Table 1), which almost covers the entire distribution range of this species. Four individuals of *Oratosquilla kempi* were also collected from South China Sea and used as out-group for phylogenetic analyses. Specimens were preserved in 95% ethanol for subsequent analysis.

Total genomic DNA was extracted from abdominal muscle using a DNeasy tissue kit (Qiagen) following the manufacturer’s protocol. The 5’ end of the mtDNA COI gene (658 bp) was amplified for all samples following cheng *et al*.^79^ with the universal primers LCO1490 and HCO2198^80^. Considering the extensive sampling and patterns revealed by mtDNA COI in coastal waters of China, the nrDNA ITS spacer (ITS1-5.8S-ITS2) was amplified for a subset of samples (14 localities, 147 specimens) following Larsen^81^ with the primers ITS1 and ITS4^82^. PCR products were purified using the QIA-quick gel purification kit (QIAGEN), in accordance with manufacturer’s instructions. The purified PCR products for mtDNA COI were used as template for direct sequencing on an ABI Prism 3730 (Applied Biosystems) automatic sequencer. Sequencing reactions were performed on both forward and reverse strands. Direct sequencing of nrDNA ITS region yielded unreadable sequence data due to intra-genomic variation. In this case, the purified PCR products were cloned with the pMD19-T Vector Cloning kit (Takara Biotechnology). Positive colonies were subjected to direct PCR with the M13 primers using the same PCR conditions for nrDNA ITS. More than three clones were picked for each individual that showed incongruence between mitochondrial and nuclear markers in the primary analysis.

Sequences were assembled in MEGA v6.06^83^, aligned with Muscle default settings and further refined manually. The nrDNA ITS had highly variable regions and the alignments needed introducing several gaps (insertions/deletions). In this case, regions with adjacent mononucleotide or microsatellite repeats were excluded because uncertainty of homology could be exacerbated by potential inaccuracies of enzymatic processes during PCR amplification and sequencing^84, 85^. Indels introduced into the alignment were coded as binary characters following the simple insertion/deletion coding method^47^ using FastGap v1.2^86^.

We used multiple recombination detection methods implemented in RDP4^87^ to identify putative recombination events between the nrDNA ITS sequences. Seven methods (RDP, Chimaera, BootScan, 3Seq, GENECONV, MaxChi and SiScan) were applied to minimize the risk of false positives. The threshold *P*-value was set at 0.05 using Bonferroni correction for multiple comparisons.

### Phylogenetic analyses

MtDNA COI-inferred haplotype phylogenies were constructed using maximum-likelihood (ML) analysis and Bayesian inference (BI) implemented in MEGA v6.06 and MrBayes v3.2.6^88^, respectively. The haplotypes from *O. Kempi* were used as out-group. The best-fitting substitution model was determined using the program jModeltest^89^ under Akaike’s information criterion. The ML analysis was conducted under the chosen substitution model (TrN+I+G) with the Nearest-Neighbour Interchange tree-swapping operation. The starting tree was estimated by BioNJ. The robustness of the ML topology was tested with 1000 bootstrap replicates. For Bayesian analyses, two independent runs were carried out for 30 million generations with a sampling frequency of 1000. Convergence was assessed by monitoring average standard deviations of split frequencies between two simultaneous runs (<0.01) and potential scale reduction factor (PSRF, close to 1.0). The program Tracer v1.6^90^ was applied to check all parameters for effective sampling size and unimodel posterior distribution. The first 25% of sampled trees were discarded as burn-in and the posterior probabilities were calculated from the remaining trees. In addition, genealogical relationships among haplotypes were assessed using a minimum spanning tree constructed by ARLEQUIN V3.5^91^.

To test the concordance across nuclear and mitochondrial markers, a subset of specimens (n = 147) was sequenced for nrDNA ITS and phylogenetic analyses were performed under the same settings described above. The phylogenetic trees were unrooted because no suitable out-group was available owing to numerous ambiguous segments during alignments with other available stomatopod nrDNA ITS sequences. For Bayesian analyses, the best fitted model and its associated parameters were estimated separately for DNA and gap data partitions. The GTR+I+G model was chosen for DNA data, and the restriction site (binary) model with variable coding bias was applied for the gap characters assuming that the rates varied over such positions according to a gamma distribution. For ML analysis, the gaps were treated as missing data.

MEGA v6.06 was explored to calculate pairwise sequence divergences (uncorrected *p*-distances) between and within lineages as indicated by the phylogenetic analyses. We applied the species criterion (“4 × rule”) to delimit cryptic species^46^, which states that monophyletic lineages represent independent evolutionary units when the mean sequence difference between lineages is more than four times greater than the average variation within the lineage.

### Population structure and diversity

Considering small sampling size around Hainan Island, samples from four sites (XY, PQ, YGH and SYS) in South China Sea were grouped as a single population (HN) after confirming non-significant between-site differentiation. Population-level pairwise genetic divergence was evaluated by the fixation index *F*_ST_^92^. The significance of *F*_ST_ was tested with 1000 permutations after sequential Bonferroni adjustments. Furthermore, we conducted a hierarchical analysis of molecular variance (AMOVA) to estimate population structure among putative regional grouping according to the structured haplotypes and *F*_ST_ values between regions. For all calculations, significance was assessed by 1000 permutations and *P* values from multiple comparisons were Bonferroni-adjusted. All genetic structure calculations were performed in ARLEQUIN v3.5. Molecular diversity was estimated by the number of haplotypes (*n*), haplotype diversity (*h*) and nucleotide diversity (*л*) for each population and each lineage with ARLEQUIN, and confidence intervals were calculated with 1000 permutations.

### Divergence time estimation

Bayesian molecular dating method was employed to estimate the divergence time between *O. oratoria* mtDNA lineages implemented in BEAST v.1.7.5^93^. Prior to the analysis, we tested the null hypothesis of equal molecular clock rate between lineages using Tajima’s relative rate test^94^ implemented in MEGA v6.06. Given that the result failed to reject the clock model (*P* > 0.5), we used a strict clock model and a coalescent tree model with constant population size in the BEAST analysis. As no mutation rates have been reported for stomatopods, this uncertainty was taken into our analyses such that a range of mutation rates (1.4–2.33% per million years estimated for crustacean species^95, 96^) was used as an a prior uniform distribution of the mutation rate of COI in BEAST to get a rough estimate of divergence time. We performed three independent runs in BEAST with a Markov chain Monte Carlo (MCMC) chain length of 10 million generations and sampling every 1000 generations. Three runs were then combined with logCombiner v1.7.5^93^ and the first 1 million generations of each run were discarded as burn-in. The program Tracer v1.6^90^ was used to ascertain that analyses converged to similar posterior probabilities and that the effective sampling size for each parameter exceeded 200. Node ages and lower bounds of the 95% highest posterior density intervals for divergence time were calculated using TreeAnnotator v1.7.5^93^ and visualized in FigTree v1.4.1^93^. While the assumption underlying the molecular clock approach in estimating divergence time was called into question^97^, we would regard time estimates as approximations on the scale of geological eras.

### Demographic inference

Several alternative methods were used to examine the historical demography of *O. oratoria*. First, Tajima’s *D* test^98^ and Fu’s *F*_S_ test^99^ were calculated to test for neutrality. Significant negative *D* and *F*_S_ statistics can be interpreted as signatures of population expansion. Second, historical demography was further investigated by examining the frequency distributions of pairwise differences between sequences (mismatch distribution). The distribution is usually unimodel for lineages following a recent bottleneck or population expansion and multimodel in samples drawn from populations at demographic equilibrium. In addition, we tested the goodness-of-fit of the actual distributions with the expected distributions under a sudden expansion model by calculating the sum of squared deviations (SSD) and Harpending’s raggedness index (RI) following 1000 coalescent simulations. Both neutrality tests and mismatch analysis were performed in ARLEQUIN. Third, we conducted Bayesian skyline plots (BSPs) with BEAST v.1.7.5 to infer past demographic changes using 50 million MCMC steps, sampled every 1000 generations under the assumption of a strict clock and the nucleotide substitution model inferred with jModeltest. Tracer v1.6 was used to check the convergence to the stationary distribution and sufficient effective sampling sizes for each estimated parameter. After discarding the first 10% of the trees as the burn-in, the same software was then used to perform the Bayesian skyline reconstruction. The mitochondrial COI was utilized as the reference marker in the BSP analysis considering the complicated evolutionary processes of nuclear ITS^24^. We used the same substitution rate as in the previous analyses to convert the parameters to actual time quantities. These analyses were performed separately for each genetic lineage identified in this study.

## Electronic supplementary material

Supplementary Information
