# Supplementary material for: Cryptic diversity in the Japanese mantis shrimp Oratosquilla oratoria (Crustacea: Squillidae): Allopatric diversification, secondary contact and hybridization
Source: Sci Rep. 2017 May 16;7:1972. doi: 10.1038/s41598-017-02059-7 (PMC5434036; doi:10.1038/s41598-017-02059-7)
Supplement: Supplementary file 1 — Supplementary Information [file 41598_2017_2059_MOESM1_ESM.pdf]

Cryptic diversity in the Japanese mantis shrimp *Oratosquilla oratoria* (Crustacea: Squillidae): Allopatric diversification, secondary contact and hybridization

Jiao Cheng & Zhong-li Sha*

Institute of Oceanology, Chinese Academy of Sciences, Qingdao 266071, China

*Corresponding author：E-mail: shazl@qdio.ac.cn

Table S1 Sampling, mtDNA COI haplotype and nrDNA ITS ribotype information for putative hybrid individuals.

			mtDNA COI		nrDNA ITS	
Sample locality	Abb.	Specimen ID	mtDNA COI haplotype	mtDNA COI lineage	nrDNA ITS lineage	No. of clones (no. of ribotypes)
Ariake Sea	AS	AS4	Hap133	S	N&S	4 (3)
		AS10	Hap117	S	N&S	4 (4)
Setonaikai	SE	SE4	Hap2	S	N&S	2 (2)
		SE5	Hap145	S	N&S	3 (3)
		SE16	Hap138	N	N&S	3 (3)
Aichi	AI	AI1	Hap1	S	N&S	3 (3)
		AI20	Hap11	N	N&S	3 (3)
Ishikawa	IS	IS1	Hap201	S	N&S	2 (2)
		IS2	Hap23	S	N&S	3 (3)
		IS7	Hap208	S	N&S	3 (3)
		IS9	Hap209	S	N&S	4 (4)
		IS11	Hap202	S	N&S	3 (2)
		IS14	Hap108	S	N&S	3 (3)
		IS15	Hap205	N	N&S	2 (2)
		IS17	Hap135	S	N&S	3 (2)
		IS18	Hap2	S	N&S	6 (5)
		IS19	Hap184	N	N&S	7 (6)
Aomori	AO	AO1	Hap2	S	N	10 (10)
		AO4	Hap14	S	N	10 (4)
		AO19	Hap195	S	N	8 (5)

Table S2 Pairwise F_{ST} comparisons between different sampling locations under the same or different lineages inferred from mtDNA COI (below diagonal) and nrDNA ITS (above diagonal).

	Lineage N													Lineage S												
Locality	QHD	LYG	SY-N	DL	DY	QD	DF-N	OT	AO-N	IS-N	AI-N	SE-N	AS-N	DF-S	SS	NJ	XM	BH	IS-S	AI-S	SE-S	AS-S	AO-S	SY-S	MI	HN
QHD	—																									
LYG	-0.001	—																								
SY-N	0.003	-0.008	—																							
DL	0.004	0.004	-0.006	—	-0.020	-0.014	0.009	0.041	-0.017	-0.006	0.014	0.139	0.084	**0.756****	**0.720****	**0.759****	**0.771****	**0.742****	**0.750****	**0.787****	**0.781****	**0.780****				
DY	-0.001	0.008	-0.012	0.009	—	-0.002	0.017	0.047*	-0.004	0.017	0.046*	0.106	0.041	**0.778****	**0.741****	**0.778****	**0.787****	**0.774****	**0.772****	**0.800****	**0.793****	**0.795****				
QD	-0.006	0.002	-0.008	-0.009	0.020	—	0.003	0.017	-0.010	-0.010	0.025*	0.111	0.068	**0.778****	**0.737****	**0.778****	**0.788****	**0.774****	**0.771****	**0.805****	**0.795****	**0.798****				
DF-N	-0.030	-0.011	-0.019	-0.009	-0.013	-0.006	—	0.031*	0.015	0.001	0.030*	0.109	0.044	**0.779****	**0.740****	**0.779****	**0.789****	**0.775****	**0.772****	**0.804****	**0.795****	**0.798****				
OT	-0.002	0.017	0.000	0.019	-0.012	0.025*	-0.001	—	0.016	0.016	0.011	0.059	0.184*	**0.791****	**0.744****	**0.791****	**0.800****	**0.791****	**0.784****	**0.821****	**0.806****	**0.815****				
AO-N	0.040*	0.031*	0.013	0.016	0.043	0.010	0.038	0.032*	—	0.009	0.040*	0.088	0.072	**0.791****	**0.754****	**0.79****	**0.799****	**0.791****	**0.785****	**0.811****	**0.803****	**0.807****				
IS-N	0.001	0.028	0.038	0.048*	0.006	0.057*	-0.004	0.005	0.067*	—	0.035*	0.080	0.014	**0.781****	**0.748****	**0.780****	**0.789****	**0.779****	**0.776****	**0.798****	**0.793****	**0.794****				
AI-N	-0.034	-0.040	-0.063	-0.055	-0.063	-0.04	-0.071	-0.054	-0.009	-0.034	—	-0.060	-0.017	**0.708****	**0.690****	**0.708****	**0.718****	**0.688****	**0.702****	**0.712****	**0.725****	**0.712****				
SE-N	-0.014	-0.032	0.022	0.021	0.020	-0.014	-0.059	0.044	0.023	-0.036	0.043	—	0.201	**0.710***	**0.679****	**0.718****	**0.732****	**0.671***	**0.706***	**0.738***	**0.745***	**0.734***				
AS-N	0.087	0.101	0.226	0.226	0.215	0.129	0.018	0.254	0.190	-0.076	0.351	-0.335	—	**0.730***	**0.702****	**0.737***	**0.751****	**0.694***	**0.725***	**0.758***	**0.764****	**0.752***				
DF-S	**0.904****	**0.909****	**0.915****	**0.919****	**0.910****	**0.912****	**0.899****	**0.917****	**0.920****	**0.895****	**0.910****	**0.901***	**0.899**	—	0.035	-0.002	0.008	-0.011	0.035	0.025	-0.005	0.017				
SS	**0.901****	**0.906****	**0.911****	**0.914****	**0.906****	**0.909****	**0.897****	**0.913****	**0.915****	**0.894****	**0.904****	**0.897***	**0.894***	-0.021	—	0.013	0.016	-0.007	0.050*	0.057*	0.044*	0.038				
NJ	**0.910****	**0.913****	**0.918****	**0.921****	**0.914****	**0.916****	**0.908****	**0.920****	**0.923****	**0.905****	**0.916****	**0.911***	**0.909***	-0.008	0.014	—	-0.027	-0.016	0.044*	0.030	-0.002	0.011				
XM	**0.913****	**0.917****	**0.923****	**0.926****	**0.918****	**0.920****	**0.911****	**0.924****	**0.928****	**0.907****	**0.924****	**0.918***	**0.917***	0.001	0.024	0.011	—	-0.007	0.065*	0.070*	0.015	0.028				
BH	**0.910****	**0.915****	**0.921****	**0.925****	**0.915****	**0.918****	**0.906****	**0.922****	**0.926****	**0.902****	**0.920****	**0.913***	**0.911**	-0.020	0.003	-0.021	-0.011	—	-0.011	-0.003	-0.022	-0.032				
IS-S	**0.915****	**0.920****	**0.927****	**0.931****	**0.921****	**0.923****	**0.913**	**0.929****	**0.933****	**0.908****	**0.935****	**0.929***	**0.930**	-0.017	0.005	-0.027	-0.001	-0.039	—	0.020	0.002	-0.033				
AI-S	**0.905****	**0.911****	**0.918****	**0.922****	**0.912****	**0.914****	**0.900****	**0.920****	**0.923****	**0.895****	**0.914****	**0.902***	**0.899**	-0.006	-0.002	-0.011	0.032	-0.017	-0.013	—	0.016	0.006				
SE-S	**0.914****	**0.917****	**0.922****	**0.925****	**0.918****	**0.920****	**0.912****	**0.923****	**0.927****	**0.909****	**0.921****	**0.917***	**0.915***	0.004	0.006	0.022	0.068*	0.034	0.017	0.008	—	-0.027				
AS-S	**0.909****	**0.913****	**0.918****	**0.921****	**0.913****	**0.915****	**0.906****	**0.919****	**0.922****	**0.903****	**0.915****	**0.909***	**0.907**	0.008	0.030*	0.001	0.021	-0.010	-0.019	-0.015	0.029*	—				

AO-S	**0.907****	**0.915****	**0.924****	**0.929****	**0.916****	**0.919****	**0.899***	**0.926****	**0.931****	**0.890***	**0.935***	**0.914**	**0.918**	-0.063	-0.041	-0.032	-0.068	-0.082	-0.011	-0.049	0.038	-0.051	—				
SY-S	**0.906**	**0.915**	**0.924**	**0.930**	**0.916**	**0.919**	**0.894**	**0.927**	**0.933**	**0.883**	**0.942**	**0.904**	**1.000**	-0.240	-0.289	-0.082	-0.268	-0.150	0.064	-0.130	0.048	-0.051	-0.425	—			
MI	**0.917****	**0.920****	**0.925****	**0.928****	**0.921****	**0.922****	**0.916****	**0.927****	**0.930****	**0.912****	**0.928****	**0.923***	**0.922**	-0.017	-0.009	-0.008	0.008	-0.015	-0.002	-0.020	0.023	0.002	-0.053	-0.113	—		
HN	**0.914****	**0.917****	**0.922****	**0.925****	**0.918****	**0.919****	**0.912****	**0.923****	**0.926****	**0.909****	**0.922****	**0.918***	**0.916**	-0.015	-0.014	-0.001	0.015	-0.015	-0.004	-0.017	0.030	0.015	-0.032	-0.192	-0.018	—	

$^{*}P < 0.05$; $^{**}P < 0.001$
